# Developing consensus for postgraduate global health electives: definitions, pre-departure training and post-return debriefing

**DOI:** 10.1186/s12909-016-0675-4

**Published:** 2016-06-04

**Authors:** Eva Purkey, Gwendolyn Hollaar

**Affiliations:** Department of Family Medicine, Queen’s University, 220 Bagot Street, Kingston, Ontario K7L 5E9 Canada; Global Health and International Partnerships, University of Calgary, Dean’s Office, 7th Floor, Teaching Research & Wellness Building, 3330 Hospital Drive NW, Calgary, Alberta T2N 4N1 Canada

**Keywords:** Global health, International education/training, Medical education, Developing/underdeveloped nations

## Abstract

**Background:**

Global health (GH) electives are on the rise, but with little consensus on the need or content of pre-departure training (PDT) or post-return debriefing (PRD) for electives in postgraduate medical education.

**Methods:**

Using a 2-iteration Delphi process to encourage discussion and consensus, participants from 14 medical schools across Canada provided input to promote more uniform policy towards defining GH electives, when PDT and PRD should be mandatory and what curriculum should be included.

**Results:**

There is consensus that PDT and PRD should be mandatory for international electives. Respondents felt that PDT should include a broad range of topics including objectives, travel safety, personal health, logistics, ethics of GH, scope of practice/supervision, and cultural awareness. PRD should include elective evaluation, lessons learned, knowledge translation, review of health and safety, and issues of reintegration. The format of PDT and PRD needs to be individualized to each institution to fit within the limitations of faculty who can serve as facilitators. Global health educators agreed on the importance of mandatory PDT and PRD for remote Canadian aboriginal electives, but did not feel that they could make recommendations without additional input of aboriginal scholars.

**Conclusions:**

All residency programs that send residents on international electives should work towards instituting quality, mandatory PDT and PRD. PDT and PRD should be recognized by universities as having academic merit and by program directors as core resident learning activities. Curriculum and objectives could be arranged around CanMEDS competencies, a physician competency framework that emphasizes qualities beyond medical expert such as professionalism, health advocate, and collaborator.

## Background

Numbers of medical students and residents participating in global health (GH) electives steadily increases [1]. The benefits for medical students experiencing global electives include an increased likelihood that they will care for underserved populations, be involved in primary care, have interest in humanitarianism and public health, be sensitive to cultural and linguistic differences, and have greater appreciation for health advocacy [2, 3]. Some of the ethical concerns for GH electives include health risks, negative impact to local patients from language/cultural barriers or limited skill levels, increased time demands on host preceptors, and financial burdens to host institutions [4–8].

The Liaison Committee on Medical Education (LCME), the accrediting body for medical education programs in the United States and Canada, recently recognized the responsibility of medical schools to improve the health and safety of students, patients and host communities, by preparing students for global health experiences [9]. LCME has mandated pre-departure training (PDT) in the accreditation review of medical schools. The growth of global health experiences amongst students and the recent accreditation standard set by LCME speaks to the importance of this issue.

Medical students in Canada working towards their MD are termed “undergraduate” despite the fact that most programs require a completed, or near completed, undergraduate degree prior to beginning medical education. It is this “undergraduate” medical education that the LCME recommendations address. After completing an MD, graduates apply to residency programs, considered “postgraduate”. Physicians in Canada cannot practice independently without having completed residency training, which lasts a minimum of 2 to 5 years and can be in a wide variety of fields ranging from family medicine and general pediatrics to various medical and surgical subspecialties. Currently there are no formal recommendations in Canada in postgraduate medical education regarding PDT for GH electives. This is an issue of increasing concern for GH educators across the country. It is this gap in guidelines and curriculum for postgraduate medical learners (residents), along with the perceived lack of importance given by post-graduate medical education to preparing residents for GH electives that this project sought to address.

Postgraduate medical education has similar trends to undergraduate medical education with increasing numbers of residents from a wide variety of specialties participating in international medical electives (IME). A recent survey reports that the percentage of residents who have interest in IME ranges from 55 to 98 % [10]. Yet while there is a great body of literature addressing IME at the undergraduate medical level, there is very little on PDT for postgraduate learners doing IME. Only one article described PDT, focusing primarily on safety concerns for residents [11], while others identified PDT as an essential component for IME, but articulated problems including lack of standardization for these electives or for PDT [12, 13].

‘Global health electives’ is a term that is increasingly being used; it includes IME, but is broader in its scope. Resident Doctors of Canada produced *Guidelines for resident physician participation in GH elective placements* and defined GH electives as experiences in under-resourced or marginalized populations locally, nationally, and abroad. In this document they provide an outline of topics that PDT should cover [14]. Likewise, the Canadian Association of General Surgeons has a checklist for residents going on GH electives, including licensing and personal health and safety issues [15]. Discussions with Canadian postgraduate medical education faculty prior to beginning this project, confirmed that PDT varies widely among and within university residency programs. Pathways for elective approval, PDT, and evaluation of the experience are not well established.

If PDT is mandatory in undergraduate medical education, it is not clear why it is not mandated at the postgraduate level. Postgraduate medical educators should share equal concern as their undergraduate counterparts with learner health and safety issues. The literature identifies many themes and concerns. Postgraduate learners, who have more clinical competence and exposure to more complex clinical problems, will likely encounter greater ethical dilemmas than medical students whose role is more observational [16]. Ethical issues such as insufficient supervision, burden of learners to host communities, learning on patients living in poverty, disparate healthcare services for rich and poor have equal relevance at the postgraduate level [17, 18]. Like medical students, residents should address these issues in a mentored environment prior to the elective. They should have some public health perspectives and understand the sociocultural and political environment of their destination. PDT should not be limited to personal health and safety, but include discussions of ethics, colonialism, power, and human dignity [19, 20]. These discussions can enhance resident recognition of ethical issues and minimize the risk of doing harm.

GH competencies are receiving increased attention at national and international levels. The World Federation for Medical Education suggests that medical education, including postgraduate, should produce “global health practitioners”, those who think globally while acting locally, in whatever country they may be, and who can uphold principles of global social responsibility and accountability [10, 21]. Competencies in global health are not uniformly listed, but typically refer to an “understanding of global burden of disease, travel medicine, healthcare disparities between countries, immigrant health, primary care within diverse cultural settings, and skills to better interface with different populations, cultures, and health systems” [22]. Offering GH clinical experiences in residency can contribute to a physician workforce able to care for mobile, heterogeneous and culturally diverse societies [19]. By teaching residents GH principles and providing mentored GH experiences, local communities will benefit from these experiences as well.

With this background, our study sought to answer 4 questions: (1) what constitutes a GH elective; (2) should there be mandatory pre-departure training (PDT) and/or post-return debriefing (PRD) for GH electives in residency in Canada; (3) what should the core content of PDT/PRD curriculum be; and (4) how should it be delivered.

## Methods

**Fig. 1:**
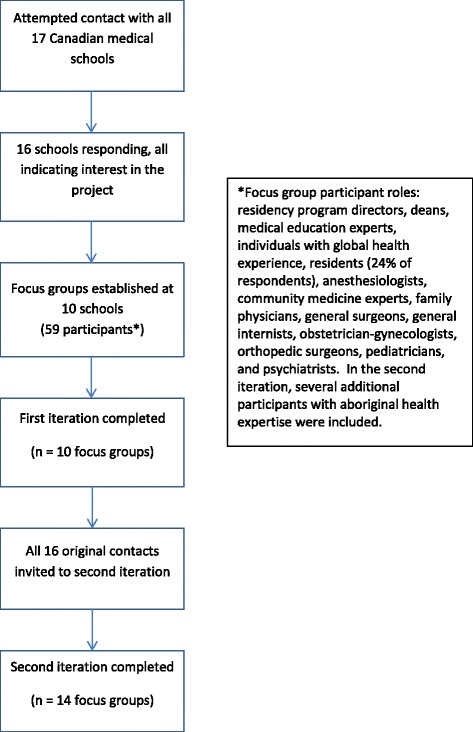
Participant sampling, representation, and response rate

We used a modified Delphi consensus process starting in the spring of 2014 to answer the above questions. We approached global health educators from each of the 17 medical schools with post-graduate programs in Canada. A site contact was established at 16 schools, and they were asked to establish and lead a local focus group. Resident Doctors of Canada was approached and elected to have members participate at the level of their school. A discussion guide was circulated to site contacts to be completed either as an online document through FluidSurvey or as a word document. Ten schools completed the first iteration (Fig. 1).

The first Delphi iteration focused on the definition of a GH elective, existing availability of PDT/PRD in post-graduate training programs at each university, level of consensus around the need for PDT/PRD, how PDT/PRD should be administered, and what topics should be covered. The group discussion guide was divided into 3 sections (Appendix 1). For the discussion of PDT/PRD content, groups were given the AFMC list of topics (personal health, travel safety, cultural competency, language competencies, and ethical considerations) as a starting point [23].

Responses from section 1 were analyzed quantitatively. Investigators analyzed responses from sections 2 and 3 thematically and summarized the information into summary statements. The aim of the 2nd iteration was to summarize and articulate the themes from the first iteration and thereby validate the analysis. The 2nd iteration had five sections (Appendix 1); it consisted of summary statements to which group participants could agree or disagree and provide reasons for disagreement. This information was sent to the 16 original site contacts for the second iteration. Fourteen schools responded to this second iteration.

At the beginning of the study, site contacts were advised three iterations might be required. However consensus was sufficiently high after the 2nd iteration that this was deemed unnecessary by investigators.

The introduction in the discussion guide provided rationale for the study. This also informed Delphi group participants that accepting to participate in the discussion implied consent with reassurances about confidentiality. Site contacts were asked only to submit the roles of their group participants for confidentiality. Ethics approval was obtained through the Health Sciences and Affiliated Teaching Hospitals Research Ethics Board of Queen’s University in Kingston, Ontario prior to the study.

## Results

### Current state and consensus of PDT and PRD for resident electives

#### Definition of global health electives

The 1st iteration identified GH electives to include the following: IME in high-income countries, IME in low and middle-income countries (LMIC), aboriginal health electives in Canada, electives working with vulnerable populations in Canada (i.e. inner city poor, homeless, prisoners, immigrants and refugees) and research-oriented electives in the above settings. Groups suggested that inequity was the fundamental issue in defining a GH elective within Canada. For the 2nd iteration, GH electives were divided into 3 broad categories, namely IME, aboriginal electives within Canada (predominantly remote), and other vulnerable population electives within Canada.

#### Current state of PDT and PRD programs

**Fig. 2:**
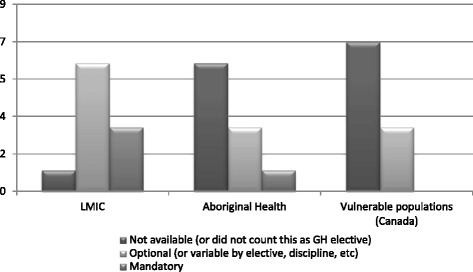
Existing availability of PDT for residents going on GH electives at 10 participating medical schools as of July 2014 (1st iteration). Note that numbers do not add up to 10 as respondents may have cited more than one elective, or no electives, in a given category

**Fig. 3:**
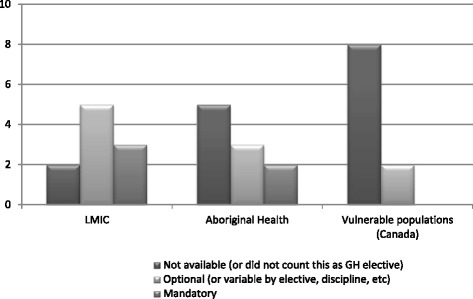
Existing availability of PRD for residents going on GH electives at 10 participating medical schools as of July 2014 (1st iteration). Note that numbers do not add up to 10 as respondents may have cited more than one elective, or no electives, in a given category

The offering of PDT varies between institutions depending upon the type of GH elective (Fig. 2). Certain institutions, for example, have mandatory PDT for residents participating in IME, while others vary by department, institutional commitment (funding, participation in pre-existing international elective partnerships), or destination (low-middle income country (LMIC) versus high income country). There is a similar range in the availability of PRD for GH electives, again ranging from unavailable to mandatory, closely mirroring the availability of PDT at participating schools (Fig. 3).

#### Ideal state of PDT and PRD for IME

Among sites, there was universal consensus that PDT should be mandatory for all residents traveling on IME (Appendix 2). While the 1st iteration of the Delphi identified some opinion differences for mandatory PDT for IME in LMIC versus high-income countries, participants in the 2nd iteration were in 100 % agreement with mandatory PDT for all IME. Thus the difference between LMIC versus high-income country electives appeared to be a distracting factor in comparison to the importance of PDT as a whole. There was a 93 % consensus among universities that PRD should also be mandatory for IME. These results reflect a gap between existing policy and practice of institutions and the ideals of respondents (Figs. 2 and 3; Appendix 2).

#### Ideal state of PDT and PRD for aboriginal health electives

The 2nd iteration demonstrated 100 % consensus on mandatory PDT and 86 % consensus on mandatory PRD for aboriginal health electives (Appendix 2). In current practice, however, there is very little optional or mandatory PDT or PRD available for aboriginal health electives (Figs. 2 and 3).

Although some institutions identified GH electives to include aboriginal electives in the 1st iteration, there is historical complexity within Canada in labeling electives within remote aboriginal communities as GH electives. Instead of focusing on the label, we attempted to use the 2nd iteration to delve deeper into opinions on content and delivery of PDT and PRD for these electives. Groups were asked, if possible, to seek additional input from aboriginal scholars when completing the 2nd iteration. Focus group participants were aware that their opinions on content and delivery for aboriginal health electives would be reported only with consent and participation of the Indigenous Physicians Association of Canada (IPAC). It became clear during this 2nd iteration that an in-depth discussion of aboriginal health programming was beyond the scope of this project. More attention and time needs to be devoted specifically to establishing guidelines for aboriginal health elective PDT/PRD due to the historical complexity of Canadian aboriginal people, the limited aboriginal health curriculum in undergraduate medical education, inadequate expertise within the groups of respondents, and lack of official participation from IPAC. As such, the discussion regarding PDT and PRD for aboriginal health electives will not be reported here, except to say that substantially better preparation was recommended by group participants than what is currently offered.

#### Ideal state of PDT and PRD for other vulnerable population electives within Canada

As with aboriginal health electives, availability of PDT/PRD for electives involving Canadian vulnerable populations was minimal (Figs. 2 and 3). There was no consensus whether PDT and PRD should be mandatory or optional for these electives. It was suggested by some groups that all Canadian physicians will interact with persons from vulnerable populations and as such, cultural skills and awareness necessary to effectively care for such populations within Canada, should be mandatory core teaching in all residency training programs. Consensus was not sought for this type of elective, but investigators encouraged group participants to bring this discussion back to their residency training programs.

### Core topics for PDT and PRD

A broad list of PDT core topics was collected during the 1st iteration (Table 1). In the 2nd iteration, no one wanted to remove any of the core topics. Only one group elected to add a topic, but this was felt by investigators to fall within the scope of an existing topic. There was no consensus on the ranking of these topic areas by respondents. Patterns were few, with the exception that objectives, ethics, cultural awareness, and scope of practice were ranked as slightly more important, and language competency as comparatively less important than other topics. Perhaps the lack of consensus was best illustrated by the comment of one group stating “[they] reject ranking” viewing all listed topics as important or core. In whatever manner this content is delivered and wherever a particular program’s emphasis lies, respondents agree that all of these core topics should be covered to some degree.

**Table 1:** Core topics for pre-departure training (2^nd^ iteration)

1. Objectives (motivations and expectations)
2. Travel safety (risk management, emergency contacts, travel advisories, embassies, etc.)
3. Personal health (health insurance, protective equipment and post-exposure prophylaxis, travel medicine, psychological adjustment)
4. Logistics (licensing and insurance, travel, transportation and accommodation)
5. Knowledge of country of destination (culture and politics, health systems organization)
6. Global health and development concepts
7. Ethics of global health
8. Scope of practice and supervision
9. Cultural awareness
10. Language competency
11. Medical expert knowledge of country of destination (epidemiology, health systems organization, WHO best practices guidelines for working in underserved countries)

PRD elicited a shorter list of key topics among respondents (Table 2). As with PDT, there was no single topic that emerged as universally more important than any other. Overall, evaluation of the elective and “experience” was ranked as somewhat more important. No one ranked knowledge translation as the number one priority and review of health and safety ranked comparatively low. Again, respondents did not add any new topic areas, and no one suggested removing any of the existing ones.

**Table 2:** Core topics for post-return debriefing (2^nd^ iteration)

1. Evaluation of elective (objectives, supervision/learning, accommodation, value of PDT, suggestions for improvement)
2. Experience (ethical issues encountered, changes in perspective, things learnt, successes, failures, frustrations)
3. Knowledge translation (impact on future career planning, advocacy/reciprocity with location of elective, deliverables for host or foreign colleagues)
4. Review of health and safety
5. Reintegration

### Delivery of PDT and PRD programming

Responses in the 1st iteration for PDT and PRD programming fell into three broad categories: delivery, facilitation, and participants. Focus groups emphasized that their ideal programming suggestions need to be flexible for different environments and site appropriate.

#### PDT format: delivery/facilitation/participants

Overall there was unanimous or high consensus among university groups on the delivery of PDT (Appendix 2). Group discussions are important for shared learning, but responsibility also falls on residents to prepare themselves for the experience. PDT programs need proper evaluation to ensure they are relevant and that assigned tasks are value-added for residents. Comments provided by respondents highlighted the challenges in organizing group face-to-face PDT given off site residents, different schedules, and the relatively few GH electives from some institutions. Having protected time for PDT within residency programs (as opposed to evenings and weekends) was considered important for access and establishing its value.

While respondents felt that PDT facilitation with teaching input from a multidisciplinary team was ideal, they recognized this would be cumbersome to organize for potentially very few residents. They highlighted the importance, when possible, to include experienced residents and, for electives in Canada, community members. Most groups felt that the PDT facilitator should have GH expertise, because the bulk of PDT was cultural and ethical and not particularly clinical. But some felt that relevant clinical experience in the resident’s specialty was also important, and that this clinical lens was more important for postgraduates than for undergraduates. Finally, the group was almost evenly divided on the appropriateness of using administrative personnel for logistics and safety. Respondents felt that if administrative personnel were appropriately trained in relevant global health content and had relevant experience, they could be entrusted with certain components, or could play a “supportive role”. Respondents clearly were not comfortable with broad delegation.

Consensus was not achieved on how to group participants for PDT, particularly as it pertains to mixing training with medical students or with other health disciplines. While most agreed that people with different levels of training or from different disciplines could learn from each other about ethical and cultural challenges or safety and security issues faced in low resource settings, participants highlighted the importance of specialty-specific components and location-specific components if learners travel to different destinations. One group identified a structure in which generic material was taught to a mixed group, which was then broken down by specialty/scope of practice, and again by location/mission. The appropriateness of inserting residents into existing programs designed for medical students is an important discussion for institutions because of the mandated PDT for medical students across Canada. The responses highlighted the importance of having designated curriculum for residents as well.

#### PRD format: delivery/facilitation/participants

Similar to the PDT discussion, there was unanimous or high consensus for PRD delivery (Appendix 2). The benefit of group discussion was again recognized, but the potential for individual debriefing needed to be available for residents who identified the need and these could be single or multiple sessions. Participants agreed that PRD was time sensitive and elective experiences need formal resident evaluation that is kept on file.

Although most respondents felt global health experience was an important asset in PRD facilitation, it was also highlighted by some that PRD facilitators not be in a position to assess residents for their rotations (i.e. program directors) to enable safe and honest discussions for residents to articulate self-perceived shortcomings or possible mistakes, without fear of evaluation. Another concern for some respondents was that PRD facilitators should not have a vested interest in the elective, because it may be more difficult for residents to be critical of the elective or to have open discussion about their challenges. One group of respondents identified their experience of a group of residents who were unwilling to discuss challenges freely until the elective organizer was no longer present. PRD programmers need to recognize the tension between limited potential facilitators and the hierarchical nature of resident-preceptor relationships limiting residents’ perceived safety. All respondents agreed that facilitators should have resources available for residents who had particular difficult experiences.

All focus group participants felt that it was reasonable to debrief residents together in a group if they had travelled to the same location. Similarly all participants felt that residents should have the explicit option to have individual PRD if they preferred. Respondents who had concerns about group PRD wanted programs to pay attention to the following precautions, namely grouping residents who had somewhat similar elective experiences, concerns that some residents will not be as open in a group setting, and the potential for the discussion degenerating into “one-upmanship” about who had the better or more challenging experience. The consensus is that group PRD is reasonable, but sensitivity to individual experiences and group dynamic needs to be considered when choosing between individualized or group PRD sessions.

## Discussion

The definition of GH is evolving, but it is a broader term than international health because it includes local or international contexts where there is health inequity [24]. Aware that opinions surrounding the definition of GH electives may differ across Canada, we wanted to include all electives where universities may be providing PDT or PRD to residents. As such, the broader term of GH electives as opposed to IME, was used. The 1st iteration captured 3 broad categories that included local and international elective experiences. The 2nd iteration was then used to establish the extent to which focus group participants agreed to PDT or PRD in these contexts.

The high degree of consensus for IME lays groundwork in recommending that PDT and PRD become a mandatory accreditation standard for residency programs. Educators express challenges in preparing residents for participation in IME. These challenges include limited buy-in from non-GH educators, lack of protected time for PDT/PRD within busy residency schedules, and lack of buy-in from residents themselves when this is not deemed mandatory by their program or institution.

Similar consensus was identified towards mandatory PDT and (to a lesser extent) PRD for residents participating in remote aboriginal health electives within Canada. Given the historical realities within Canada and insufficient input from aboriginal scholars from Canadian institutions, we do not feel we can advocate for any particular PDT or PRD curriculum for these electives. Nevertheless, focus groups stressed that appropriate preparation for these electives is important. Focus groups also identified the need for core curriculum across residency training programs that enable *all* physicians who graduate from Canadian residency programs to be culturally competent when caring for aboriginal patients. The National Collaborating Centre for Aboriginal Health and the Indigenous Physicians Association of Canada drafted recommendations for cultural safety competencies for physicians caring for aboriginal patients [25]. They have also developed a set of core competencies for undergraduate medical students using the CanMEDS framework [26]. Further work is needed to explore appropriate resident preparation for those who do electives within remote aboriginal communities. Universities should also review what core aboriginal health curriculum is taught in residency training programs across specialties to determine if cultural safety competency for aboriginal patients is being taught.

The third type of GH electives includes other populations experiencing health inequities in Canada (i.e. persons living in poverty, inner city, immigrant and refugee populations). There was no consensus about PDT or PRD for resident electives focusing on these populations. Some participant groups may not have acknowledged these as GH electives, and that may have contributed to the lack of consensus. Study participants were in agreement that all physicians completing residency in Canada should have core programming during residency that equips them to appreciate the health equity barriers faced in Canada and be culturally competent to provide relevant and appropriate healthcare to these populations. Again, this highlights the opportunity for all specialties to review their resident core curriculum in addition to what is offered to those with a special interest in this area.

**Fig. 4:**
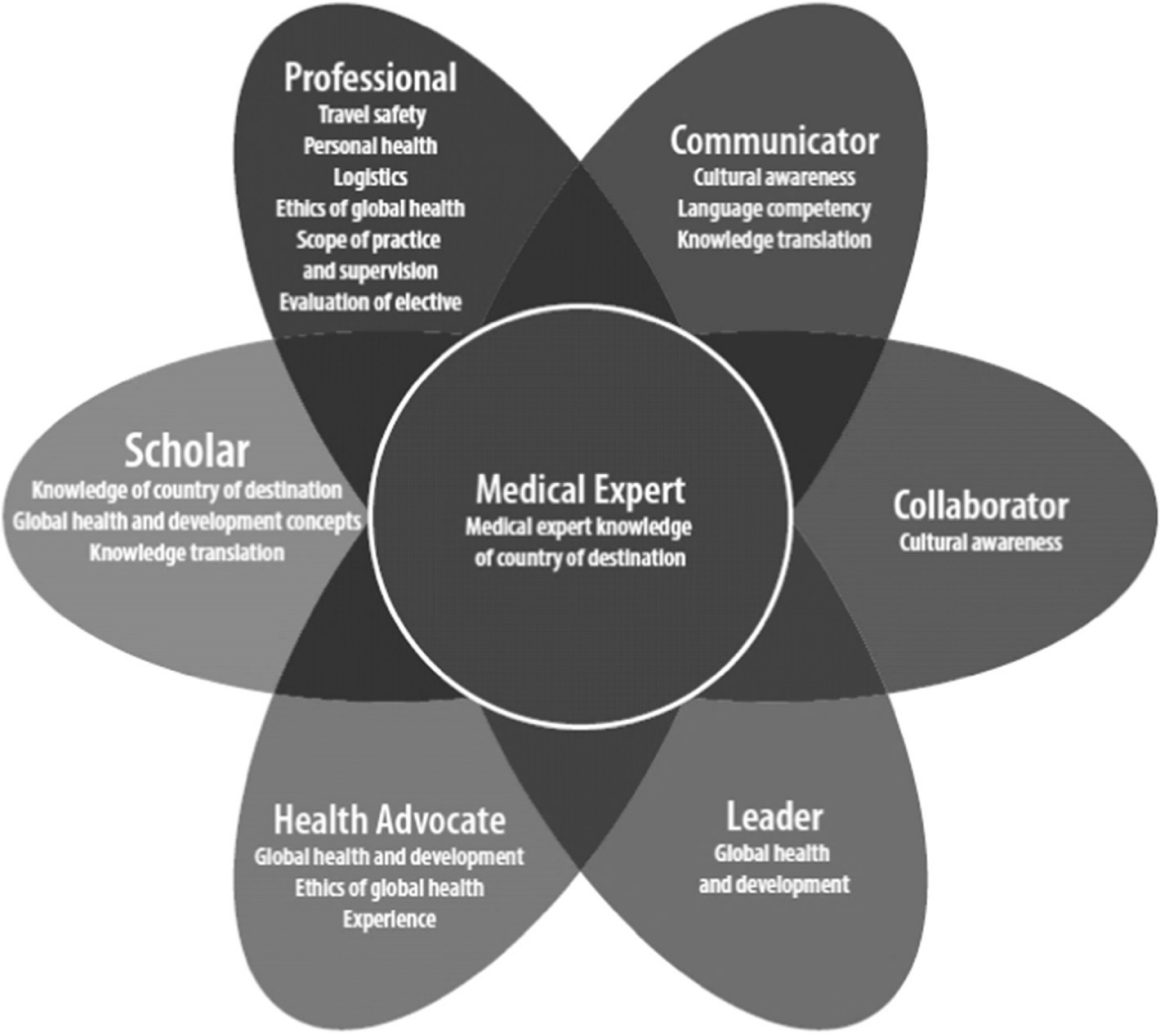
Proposed organization of pre-departure preparation and post-return debriefing objectives around CanMEDS roles. Copyright © 2015 The Royal College of Physicians and Surgeons of Canada. http://www.royalcollege.ca/rcsite/canmeds-e. Reproduced and adapted with permission

In addition to gaining consensus on PDT and PRD practice, we explored consensus on core content for IME. The original AFMC topic list for medical student PDT was not felt to be sufficient for residency training and was expanded upon. A commonly used framework for residency training programs in Canada is the CanMEDS framework (Royal College of Physicians and Surgeons of Canada). In fact this framework has been used in the past to articulate Global Health curriculum for Family Medicine residency programs [27] and for IME [28]. The seven CanMEDS roles are Professional, Communicator, Collaborator, Leader, Health Advocate, and Scholar, all around the central role of Medical Expert. Figure 4 illustrates how the core topics identified for PDT and PRD could be arranged (with some redundancy depending on how one understands each topic) around these roles. Moving forward, it may be useful to conceptualize objectives for all GH electives around these roles as well.

The AFMC guidelines do not mandate PRD for medical students. Delphi participants, however, identified PRD as an essential process for all IME, especially in LMIC. It is necessary for residency training programs to ensure that the electives provided good learning opportunities and this could be assessed in PRD. Furthermore, IME can be profound experiences emotionally and professionally, and educators need to ensure the issues encountered have a forum for discussion to help residents process their experience as part of their professional development.

Themes from the 1st Delphi iteration about PDT and PRD format were presented back to group participants as “best practices” meant to aid program developers in designing and improving their programs. The realities at universities are different, including numbers of GH faculty, numbers of interested residents, and types of electives such that mandating group PDT, or individual PRD, led by specific types of faculty would create artificial and unmanageable expectations. Delivery methods of PDT and PRD will vary, but this Delphi process has highlighted important areas for consideration when designing programs. Considerations include which learners could be prepared together, who should facilitate and the need for electives to be assessed for quality and relevance. Providing debriefing environments that are safe for residents is another important consideration.

## Conclusions

Given the degree of consensus for IME, this study leads us to recommend mandatory PDT and PRD for Canadian residents going on IME, especially to LMIC. Making this an accreditation standard at the postgraduate level would ensure this, just as it has at the medical school level. There is Canadian consensus as to what should constitute core PDT and PRD content. We recommend that this core content also become part of the accreditation standard for residency programs offering electives in LMIC. The PDT/PRD curriculum could be arranged around CanMEDS roles, as it is with other aspects of Canadian postgraduate curriculum. The results also lead us to recommend further dialogue and study regarding establishing potential PDT and PRD for electives within remote aboriginal communities and other vulnerable population groups. The results should also encourage resident training programs to assess what core curriculum they have in developing global health competencies for all physicians who finish residency, regardless of specialty. We hope this study assists in advocating for faculty time to develop excellent programs, recognizing the relevance of this work, and validating its importance by protecting resident time to attend.

## Abbreviations

AFMC, Association of Faculties of Medicine of Canada; GH, Global Health; IPAC, Indigenous Physicians Association of Canada; IME, International Medical Elective; LCME, Liaison Committee on Medical Education (LCME); LMIC, low and middle-income countries; PRD, post-return debriefing; PDT, pre-departure training.

## Appendix 1

### Overview of 2 iterations of Delphi process

**Table:** **Overview of 2 iterations of Delphi process**

First iteration	
Intro: Roles of focus group members	
Section 1: GH electives	
What constitutes a GH elective?	
Is there PDT/PRD within your institution (mandatory, optional, not available)	
Should there be PDT/PRD within your institution (mandatory, optional, not available)	
Section 2: PDT discussion	
Content and Format	
Section 3: PRD discussion	
Content and Format	
Second iteration	
Section 1: Confirmation on necessity for PDT/PRD for different types of GH electives	
(Participants given summary statements to agree or disagree and give reasons for disagreement)	
Section 2: Content of PDT	
(Confirmation of topic list and request for ranking importance)	
Section 3: Format of PDT	
(Participants given summary statements to agree or disagree and give reasons for disagreement)	
• Delivery	
• Facilitation	
• Participants	
Section 4: Content of PRD	
(Confirmation of topic list and request for ranking importance)	
Section 5: Format of PRD	
(Participants given summary statements to agree or disagree and give reasons for disagreement)	
• Delivery	
• Facilitation	
• Participants	

## Appendix 2

**Table 3:** Summary statements for 2nd delphi iteration to confirm consensus

Topic	Summary statement	Level of consensus
Necessity of PDT & PRD	PDT should be mandatory for all residents going on IME.	100 %
	PDT should be mandatory for all residents doing electives in remote First Nations communities.	100 %
	PRD should be mandatory for all residents going on IME.	High
	PRD should be mandatory for all residents doing electives in remote First Nations communities.	Mod
	PDT and PRD should be mandatory for all residents doing electives within vulnerable populations within Canada.	No
PDT Delivery	Programs are encouraged to evaluate their PDT to ensure residents feel what they are learning is relevant to their experiences.	100 %
	Individual preparatory work is appropriate, including personal research on destination and issues pertaining to resident specialty, with potential required readings and online modules.	100 %
	Group sessions are ideal, fostering team building and shared learning.	High
	Fewer longer sessions are preferable to multiple short sessions to ease delivery and resident access.	High
	Some component of PDR must be face to face (not written or online).	Mod
PDT Facilitation	There are benefits to multidisciplinary PDT.	High
	GH expertise is the most important characteristic of a PDT facilitator.	Mod
	Administrative personnel can appropriately the logistics and travel safety component of PDT.	No
PDT Participants	If a group of learners are going to the same destination, the group can involve participants from different health disciplines (i.e. nursing, physio).	Mod
	If a group of learners are going to the same destination, the group can be trained together, even if they are at different levels of training.	No
	PDT participants should be at a similar level of training.	No
PRD Delivery	A formal evaluation of the elective should be submitted to the program director and to the GH office (or similar body).	100 %
	PRD must be delivered in a safe space where residents are free to discuss difficulties and awkward situations without being judged.	100 %
	Group PRD is acceptable and may be beneficial in fostering discussion around shared experiences.	100 %
	PRD can consist of a single or iterative sessions.	100 %
	Individual PRD should be available to any resident who has experienced difficulty on the elective.	High
	Timelines are key with PRD being offered shortly after an elective.	High
PRD Facilitation	Facilitator should have resources available for residents who had difficult experiences.	100 %
	Facilitator should have knowledge and experience in GH.	Mod
	Facilitator should not be in a position to evaluate the resident.	No
	Facilitator should not have a vested interest in the elective in question.	No
PRD Participants	Learners should be offered individual PRD if they perceive this to be helpful.	100 %
	Learners who have been to the same destination can receive PRD together.	100 %
	Learners who have been to different destinations can receive PRD together if their experiences were similar.	Mod

High: High consensus is defined as 90–99 %

Mod: Moderate consensus is defined as 75–89 %

No: Less than 75 % is considered no consensus
